# Source-Free Model Transferability Assessment for Smart Surveillance via Randomly Initialized Networks

**DOI:** 10.3390/s25133856

**Published:** 2025-06-20

**Authors:** Wei-Cheng Wang, Sam Leroux, Pieter Simoens

**Affiliations:** IDLab, Department of Information and Technology, Ghent University—imec, 9052 Ghent, Belgium; sam.leroux@ugent.be (S.L.); pieter.simoens@ugent.be (P.S.)

**Keywords:** transferability assessment, unsupervised learning, smart surveillance, randomly initialized neural network

## Abstract

Smart surveillance cameras are increasingly employed for automated tasks such as event and anomaly detection within smart city infrastructures. However, the heterogeneity of deployment environments, ranging from densely populated urban intersections to quiet residential neighborhoods, renders the use of a single, universal model suboptimal. To address this, we propose the construction of a model zoo comprising models trained for diverse environmental contexts. We introduce an automated transferability assessment framework that identifies the most suitable model for a new deployment site. This task is particularly challenging in smart surveillance settings, where both source data and labeled target data are typically unavailable. Existing approaches often depend on pretrained embeddings or assumptions about model uncertainty, which may not hold reliably in real-world scenarios. In contrast, our method leverages embeddings generated by randomly initialized neural networks (RINNs) to construct task-agnostic reference embeddings without relying on pretraining. By comparing feature representations of the target data extracted using both pretrained models and RINNs, this method eliminates the need for labeled data. Structural similarity between embeddings is quantified using minibatch-Centered Kernel Alignment (CKA), enabling efficient and scalable model ranking. We evaluate our method on realistic surveillance datasets across multiple downstream tasks, including object tagging, anomaly detection, and event classification. Our embedding-level score achieves high correlations with ground-truth model rankings (relative to fine-tuned baselines), attaining Kendall’s τ values of 0.95, 0.94, and 0.89 on these tasks, respectively. These results demonstrate that our framework consistently selects the most transferable model, even when the specific downstream task or objective is unknown. This confirms the practicality of our approach as a robust, low-cost precursor to model adaptation or retraining.

## 1. Introduction

**Figure 1:**
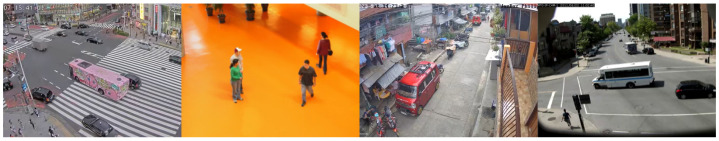
Examples of different camera configurations.

Visual surveillance is a foundational component of smart city infrastructure, supporting real-time applications such as traffic management and anomaly detection. These cameras operate under diverse configurations, varying in viewpoint, angle, elevation or location, as illustrated in Figure 1. This diversity makes it infeasible to rely on a single machine learning model that performs well across all environments. Instead, prior work has shown that camera-specific models are more effective and efficient in such settings [1,2].

Training a unique model from scratch for each surveillance camera is often infeasible due to the high computational cost and the vast amount of devices. For example, training a single model can require eight hours on a Tesla V100 GPU (NVIDIA, Santa Clara, CA, USA), and a city such as London has over 940,000 cameras [3]. An alternative strategy is to design lightweight models tailored for a specific task while reducing computational requirements [4]; such specialized models may not be readily available for all target tasks, particularly when the available data are unannotated. Consequently, a common strategy to obtain these location-specific models is to fine-tune pretrained models [5]. However, this approach is sensitive to domain shift: if the source and target domains differ significantly, model performance can degrade substantially [6]. The selection process itself is further complicated by the unreliability of reported performance metrics. For instance, a study on real-world systems found a discrepancy as high as 44% between a model’s claimed accuracy and its measured operational performance [7]. These factors highlight the critical need for methods that can estimate a model’s effectiveness on specific target data prior to deployment.

Estimating the transferability of a pretrained model to a new environment is inherently challenging as it involves quantifying the distributional shift between source and target data. In practical scenarios such as smart surveillance, access to the original training data is often restricted due to privacy concerns. While the European Union promotes increased data sharing across industrial and public sectors to drive innovation [8], the direct exchange of raw data often conflicts with GDPR [9] or risks exposing proprietary information. One solution to prevent data violation is to limit access and provide encryption on the stored data [10]. An alternative solution, the source-free setting, which shares trained models instead of data, is increasingly regarded as a privacy-preserving alternative for enabling collaboration without disclosing sensitive datasets. In addition, no labeled data are typically available for the target domain. This source-free, unsupervised setting [11] significantly complicates transferability assessment, as it precludes direct comparison between source and target domains and limits the use of traditional domain adaptation techniques.

**Figure 2:**
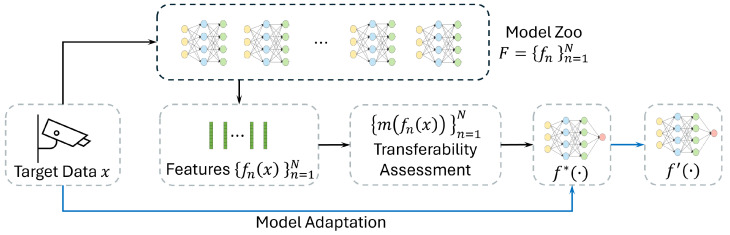
A high-level overview of source-free unsupervised domain adaptation. The black lines describe the process of transferability assessment, while the blue line describes the data flow of the model adaption. Target data is first fed into a set of pretrained models $F={\left\{f_{n}\right\}}_{n=1}^{N}$. A transferability assessment is then applied to the representations ${\left\{f_{n}\left(x\right)\right\}}_{n=1}^{N}$ to rank the applicability of each model. Finally, the highest ranked pretrained model $f^{*}\left(\cdot \right)$ is adapted to target-specific mode $f^{\prime }\left(\cdot \right)$ with the target data.

Figure 2 further illustrates this concept of source-free unsupervised domain adaptation (SFUDA) in smart surveillance settings. First, a model zoo is formed of models pretrained on data collected under diverse configurations. These models could have different architectures, training data or training procedures. When a new camera is installed in the system, a configuration procedure finds the model from the model zoo that best fits the new scene. For this, each of the models processes a small amount of camera data and the transferability assessment is used to rank them, identifying the pretrained model that is most adaptable to the target domain. Finally, the highest-ranked model is either deployed directly or after further fine-tuning on target data for a specific camera. Crucially, this procedure requires no access to the original training data of the models in the model zoo, nor does it require labeled information for the target task that will be performed on the new data (i.e., it operates in the source-free unsupervised setting).

We propose a novel approach to transferability assessment based on randomly initialized neural networks (RINNs). Unlike existing methods that rely on assumptions such as reliable model uncertainty or the generalizability of pretrained embedding spaces, assumptions which often fail in real-world surveillance scenarios, our method leverages RINNs to construct a task-agnostic, data-independent reference embedding space. Transferability is then estimated by computing the similarity between embeddings produced by each pretrained model and those from the RINN ensemble, using minibatch-Centered Kernel Alignment (CKA) for scalability and efficiency. Despite lacking training, RINNs have been shown to capture meaningful structural priors through architectural depth and compositional nonlinearity [12,13]. This enables robust model selection without requiring access to source data, target labels, or unreliable uncertainty estimates. In the context of practical applications, our framework is designed for an offline model selection phase, which would typically be executed on a server or cloud infrastructure. Following this one-time assessment, the single best-identified model could then be further fine-tuned for a specific target if needed, and subsequently deployed for efficient, real-time inference on resource-constrained edge devices at the surveillance site.

Through experimental evaluations on four unlabeled source video datasets (Tokyo Intersection [14], Tokyo Street [15], and Agdao-Market/Street [16]) and three public target sets (Street Scene [17], NWPU Campus [18], and Urban Tracker [19]), we demonstrate that this is a cost-efficient solution for source-free, unsupervised domain adaptation in smart-city environments.

To summarize, this research confronts the critical challenge of assessing model transferability under source-free and unsupervised conditions, a problem of practical importance for deploying machine learning models in real-world scenarios such as smart city surveillance. To the best of our knowledge, this specific challenge remains underdeveloped, with very few prior works addressing it directly. Our main contributions to this area are as follows:

- We introduce a novel transferability assessment framework designed for the aforementioned challenging conditions, which effectively addresses the diverse camera configurations and significant domain shifts inherent in smart surveillance applications.
- The proposed framework uniquely employs ensembles of randomly initialized neural networks (RINNs) to create a task-agnostic reference embedding space. This approach avoids biases inherent in using pretrained models for the reference itself and enables model assessment without prior knowledge of the specific downstream task or objective.
- We developed an embedding-level score ($S^{E}$) by comparing structural similarities in data representations, reflecting intrinsic data characteristics. This approach yields more robust, task-agnostic transferability estimates, avoiding the instabilities tied to pseudo label-based methods or the reliance on task similarity, particularly under shifting downstream objectives.
- Comprehensive empirical validation on three public surveillance datasets (Street Scene, NWPU Campus, and Urban Tracker, covering 48 diverse scenes) and three downstream tasks (object tagging, anomaly detection, and event classification) demonstrates our $S^{E}$ metric’s efficacy. Results confirm $S^{E}$ consistently identifies the most transferable models, proving its practical utility for identifying the most suitable model for adaptation within these challenging real-world surveillance scenarios.

The remainder of the paper is structured as follows. Section 2 introduces past related work on transferability assessment, randomly initialized neural networks, embedding similarity and source-free unsupervised domain adaptation. In Section 3, we describe the proposed framework, the construction of the reference embedding space using RINNs, and the definitions of the two novel transferability scores. The evaluation process, including the details of the model zoo, datasets, downstream tasks, and implementation specifics are also explained in Section 3. Then, the experimental results and ablation study are presented and discussed in Section 4. Finally, we conclude the findings and discuss future research directions in Section 5.

## 2. Related Work

### 2.1. Transferability Assessment

Transferability assessment provides an estimation of how a model would perform on new data. Early works estimate transferability through partial fine-tuning on the target task. While informative, this method still incurs considerable computational cost and requires fine-tuning [20]. Moreover, the need for access to annotated target data further limits the scalability of such methods in practical settings.

More efficient methods for transferability estimation methods have been proposed, each operating under varying assumptions regarding data accessibility. One of the earliest examples, Tran et al. [21], estimates task transferability via conditional entropy. However, this method assumes access to both source data and target annotations and presumes similarity between the source and target distributions. To relax the dependence on source data, several works such as Nguyen et al. [22], You et al. [20], Ding et al. [23], and Xu and Kang [24] estimate transferability by analyzing pretrained source representations applied to the target domain. The recent LEAD framework [25] proposes a transferability metric by modeling the evolution of a model’s output logits during fine-tuning. Using a single run, it captures the initial logits and their gradients to construct an ordinary differential equation (ODE), which estimates how the logits would evolve toward their final state. This theoretical trajectory is then used to assess how well the model would adapt to the target domain, which serves as an indicator for model selection. Although these methods do not need access to the source data, offering improved privacy and reduced demands on data storage and computation, they still require annotated target data. This limitation makes these methods hard to apply in real-world surveillance applications, where annotations are typically unavailable. Pei et al. [26] propose uncertainty distance (UD), which measures the transferability without accessing the source data and the annotation of the target data. Assuming a low model uncertainty, they calculate the distributional uncertainty with a probabilistic framework that leverages the source model’s predictions on target data. In contrast, Ensemble Validation (EnsV) [27] avoids dependency on a single model and instead utilizes the joint prediction from an ensemble of models in the model zoo to form a reliable proxy for the ground truth. While this ensemble-based approach provides general stability, its performance can be suboptimal if the proxy itself is unreliable, which can occur when the majority of models in the zoo were not suitable for the target data.

While these works aim to support transferability assessment in unsupervised source-free adaptation pipelines, they still rely on pretrained models and their behavior on target data. In contrast, we propose a transferability assessment that does not depend on the embedding of pretrained models. Instead, we measure the structural consistency of target data by comparing embeddings of a given model with those from randomly initialized networks.

### 2.2. Randomly Initialized Neural Network

The potential of randomly initialized neural networks (RINNs) was first highlighted by Frankle and Carbin [28] through the Lottery Ticket Hypothesis (LTH). Their work posits that dense, untrained neural networks contain sparse subnetworks which, when trained from the same initialization, can achieve performance comparable to that of fully trained networks.

Building on this foundation, Ramanujan et al. [12] demonstrated that sufficiently over-parameterized RINNs contain subnetworks that match the performance of fully trained networks without any training, a claim now referred to as the strong LTH. Malach et al. [13] provide a theoretical proof of the stronger LTH, showing that, for any bounded distribution and a target network with bounded weights, a sufficiently large RINN contains a subnetwork capable of achieving comparable performance to the target network. However, their findings also indicate that the over-parameterized RINN must be sufficiently large to satisfy these conditions, thereby imposing significant memory requirements.

To address the inefficiency caused by such over-parameterization, Chijiwa et al. [29] proposed Iterative Randomization (IteRand), a framework designed to improve parameter efficiency in pruning-based settings.

In contrast to the works described above, which focus on extracting or pruning such subnetworks from RINNs for direct deployment or fine-tuning, our approach adopts a different perspective. Rather than identifying so-called “winning tickets”, we assume that pruned or weakly activated neurons are either uninformative or adversely affected by domain shift. Consequently, our method concentrates on evaluating embedding consistency across models, without modifying or optimizing the RINN itself. This enables us to exploit the inherent structural properties of RINNs while avoiding the computational burden of subnetwork extraction or training.

### 2.3. Embedding Similarity as a Proxy for Transferability

Another important line of research investigates the direct comparison of representations between models, without considering the raw data or annotations. Prior studies have demonstrated that the representations learned by neural networks encode task-relevant structural information, and that the degree of similarity between these representations can provide meaningful insights into model behavior and transfer potential. Based on their computational mechanism,  Klabunde et al. [30] categorize these methods into six types. To estimate the similarity between different representations in our scenario, we particularly focus on the three types that meet the following two key technical requirements: the ability to compare representations with different dimensions, and the scalability to large datasets.

**Alignment-based** methods aim to find an optimal transformation that minimizes the difference between one representation and the transformed version of another. For example, Williams et al. [31] apply principles from statistical shape analysis to define novel similarity metrics on embeddings. On the other hand, **CCA-based** methods find a set of weights for each dimension of both embeddings such that the weighted embeddings have the maximal correlation. While the CCA-based methods can deal with misalignment between dimensions, they are known to suffer from high computational cost in high-dimensional settings, which can be a serious issue in smart surveillance. A recent work, Tuzhilina et al. [32], proposes several strategies to reduce this computational overhead, such as utilizing structured regularization and the kernel trick. Finally, **RSM-based** methods first compute a pairwise similarity matrix within the embedding space of each representation, where the pairwise similarity matrices of the two embeddings are then compared. A well-known method in this category is Centered Kernel Alignment (CKA) [33].

For our primary similarity metric, we adopt CKA, a powerful and widely-used benchmark shown to outperform CCA-based methods in capturing functional similarity and is flexible enough to handle representations of different dimensionalities. However, prior research also shows its limitations. Research summarized by Klabunde et al. [30] highlights that CKA can be insensitive to certain structural changes in representations and can be influenced by data manipulations that do not affect a model’s function. For instance, CKA can be insensitive to the removal of functionally important principal components and can be disproportionately affected by simple manipulations of single data points that do not change the model’s overall function. On the other hand, Hayne et al. [34] provide crucial insight here, demonstrating that CKA is a significantly better predictor of linear decodability (a proxy for available information) than it is of performance on a single, fixed network. While this may be a limitation when analyzing a single task, it is a distinct advantage for our goal of assessing general transferability. We seek to identify which pretrained embeddings are most versatile for a wide range of potential downstream applications. Therefore, a metric that effectively quantifies the richness of linearly available information is more appropriate than one tied to a single network’s function. Furthermore, given the scale of our dataset, a full-batch computation is infeasible. The minibatch-CKA proposed by Nguyen et al. [35], which uses an unbiased estimator of HSIC [36] to compute linear CKA so that the value of CKA is independent of the batch size, enables scalable assessment across large model sets and high-dimensional embeddings.

This approach enables us to assess the embedding-level consistency between a pretrained model and the structural patterns captured by RINNs, providing an efficient, label-free estimate of transferability. The use of minibatch-CKA significantly reduces both memory footprint and computational overhead. Unlike standard CKA, which requires storing and processing the full dataset to compute Gram matrices, minibatch-CKA operates on small batches and accumulates similarity statistics incrementally. This makes it particularly well-suited for evaluating large model sets on long-form or high-resolution surveillance data, enabling scalable deployment under real-world resource constraints.

### 2.4. Source-Free Unsupervised Domain Adaptation

Source-free unsupervised domain adaptation (SFUDA) is a specialized setting of adapting pretrained models to an unannotated target domain without access to the source data or to labeled target data [37]. This setting addresses real-world constraints where source data is not universally shareable due to privacy and storage limitations, conditions commonly encountered in smart surveillance scenarios. Furthermore, such a setting alleviates computational burdens, which aligns with the need for scalable and efficient deployment in edge-based systems.

While a number of methods have been proposed under the SFUDA setting [26,37,38], they primarily focus on adapting a given model to the target domain. These methods can be grouped into three broad categories: (1) self-tuning via pseudo labels and information maximization [27,37,39], (2) feature alignment using structural cues in the target domain [26], and (3) sample generation to synthesize source-like data [38].

These methods focus primarily on how to adapt the given model rather than on whether it is the right model to begin with. However, prior work [20] has shown that the choice of a good starting point can have a substantial impact on the final performance. In contrast, our work addresses this issue, proposing a transferability assessment method that operates fully within the SFUDA constraints, yet without relying on pretrained model embeddings or source data. In doing so, our method complements domain adaptation-based approaches by guiding model selection prior to adaptation, potentially improving their overall effectiveness.

## 3. Materials and Methods

In this section, we present our novel transferability assessment framework designed for source-free unsupervised domain adaptation. We first introduce the overall structure and key components of the framework. We then introduce the two proposed RINN-based assessments, label-level score $S^{L}$, and embedding-level score $S^{E}$. Finally, we describe the benchmark datasets and models that will be used in the experimental evaluation.

This proposed framework operates on video frames obtained from the target environment. While the characteristics of this data are inevitably influenced by the camera hardware, environmental conditions including lighting, and any preliminary preprocessing, the transferability assessment method itself is designed to be independent of these specific underlying hardware components, focusing instead on the data as presented.

### 3.1. Framework

**Figure 3:**
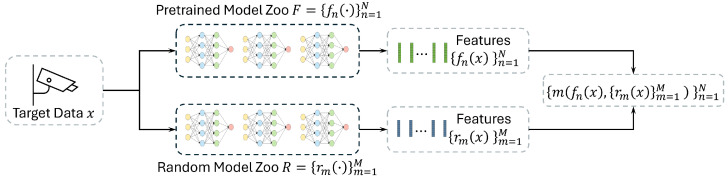
Overview of the framework. The target data is fed to both the pretrained models and the randomly initialized neural networks. We then predict the transferability of each pretrained model to the new data by estimating the mutual information between both types of representations.

Our proposed framework is illustrated in Figure 3. Let $F={\left\{f_{n}\right\}}_{n=1}^{N}$ denote a model zoo of *N* pretrained models and let *X* be the unlabelled target data. Each model $f_{n}$ encodes *X* into a representation $f_{n}\left(X\right)$; these representations are then used to evaluate the transferability of each model.

In addition, we construct an RINN zoo $R={\left\{r_{m}\right\}}_{m=1}^{M}$ based on a set of *M* randomly initialized neural networks. Given one pretrained representation $f_{n}\left(X\right)$ and the collection $R\left(X\right)={\left\{r_{m}\left(X\right)\right\}}_{m=1}^{M}$, we define a transferability score $S(f_{n}\left(X\right),R\left(X\right))$, which measures how much of the information captured by the RINNs is already present in $f_{n}\left(X\right)$. A higher score implies that $f_{n}$ is more adaptable to the target domain. We aim to rank $f_{n}$ based on their adaptability to *X* without requiring source data or target annotations. We compare two approaches of using the RINNs for transferability estimation: label-level scoring and embedding-level scoring, as explained in the following paragraphs.

#### 3.1.1. Label-Level Score

In this approach, we use the RINNs to directly generate pseudo labels Y(X) for each input *X*: $Y\left(X\right)=c_{m}^{r}\left(r_{m}\left(X\right)\right)$, where $c_{m}^{r}$ is a simple, randomly initialized classifier added on top of $r_{m}$. This layer predicts a pseudo label for each input. The predicted pseudo label has no semantic meaning but instead should be seen as an assignment to a cluster grouping similar inputs. In our experiments, we set each $c_{m}^{r}$ to predict 20 classes. We then add a similar fully-connected layer $c_{n}^{r_{m}}$ on top of each $f_{n}$ and fine-tune this layer using gradient descent to predict the pseudo label. The underlying intuition for this approach is that predicting the pseudo label is only possible if the pretrained model has a rich enough feature representation that captures broad information covering the features extracted by a large set of RINNs. The transferability score is then the sum over all the $r_{m}$:

(1) $$S^{L}\left(f_{R}\left(X\right),R\left(X\right)\right)=\sum_{M}^{m=1}\mathrm{eval}\left(c_{n}^{r_{m}}\left(f_{n}\left(X\right)\right),c_{m}^{r}\left(r_{m}\left(X\right)\right)\right)$$

where eval is a performance metric such as accuracy, F1-score or MSE.

#### 3.1.2. Embedding-Level Score

A disadvantage of the label-level score is that it requires a training step which might be costly. In addition, the pseudo labels sometimes collapse where the RINN predicts the same output for each input. A large set of RINNs is needed to avoid this issue.

An alternative scoring mechanism uses internal representations of the RINNs and pretrained models. Based on knowledge distillation approaches, we can use metrics such as correlation-based similarity [40] or Centered Kernel Alignment (CKA) to directly measure the similarities between both internal representations. The embedding level score is defined as:

(2) $$S^{E}\left(f_{n}\left(X\right),R\left(X\right)\right)=\mathrm{sim}\left(f_{n}\left(X\right),R\left(X\right)\right)$$

In this paper, we use minibatch-CKA, proposed in [35], to measure the structural similarity between $f_{n}\left(X\right)$ and R(X). We adopt minibatch-CKA not only for its alignment with representation-based analysis but also for its practical benefits: it enables scalable evaluation by computing similarity over mini-batches, thereby significantly reducing memory usage compared to standard CKA. To calculate minibatch-CKA, *X* is first divided into batches with *B* samples, $X={\left\{X_{k}\right\}}_{k=1}^{K},\left|X_{k}\right|=B$. By applying pretrained model $f_{n}\left(\cdot \right)$ and randomly initialized model $r_{m}\left(\cdot \right)$ to $X_{k}$, we obtain two representations $f_{n}\left(X_{k}\right)\in R^{B\times f_{n}^{d}}$ and $r_{m}\left(X_{k}\right)\in R^{B\times r_{m}^{d}}$. Note that $f_{n}^{d}$ and $r_{m}^{d}$ represent the number of dimensions of $f_{n}$ and $r_{m}$ which are not necessary the same. As minibatch-CKA uses a linear kernel to calculate CKA, we further define the kernel matrices $K=f_{n}\left(X_{k}\right)f_{n}{\left(X_{k}\right)}^{⊤}$ and $L=r_{m}\left(X_{k}\right)r_{m}{\left(X_{k}\right)}^{⊤}$. The minibatch-CKA is then calculated using the following equation:

(3) $${\mathrm{CKA}}^{\mathrm{mb}}\left(K,L\right)=\frac{{\mathrm{HSIC}}_{1}^{mb}\left(K,L\right)}{\sqrt{{\mathrm{HSIC}}_{1}^{mb}\left(K,K\right)}\sqrt{{\mathrm{HSIC}}_{1}^{mb}\left(L,L\right)}},$$

where

(4) $${\mathrm{HSIC}}_{1}^{mb}\left(K,L\right)=\frac{1}{k}\sum_{i=1}^{k}{\mathrm{HSIC}}_{1}\left(K_{i},L_{i}\right).$$

In Equation (3), HSIC is the Hilbert–Schmidt Independence Criterion [41], which was used to measure the dependence between two sets of variables. To make the HSIC independent to *B* so that CKA can be calculated batch-by-batch, minibatch-CKA uses the unbiased HSIC estimator (${\mathrm{HSIC}}_{1}$) in [36]. For detailed derivation and proof, please refer to [33]. With the minibatch-CKA, Equation (2) is then $S^{E}\left(f_{n}\left(X\right),{\left\{r_{m}\left(X\right)\right\}}_{M}^{m=1}\right)={\mathrm{CKA}}^{\mathrm{mb}}\left(f_{n}\left(X_{k}\right)f_{n}{\left(X_{k}\right)}^{⊤},r_{m}\left(X_{k}\right)r_{m}{\left(X_{k}\right)}^{⊤}\right)$.

### 3.2. Models

We construct a model zoo comprising models with identical architectures but trained on separate source videos. This design isolates data-induced variations in learned representations while controlling for architectural differences. All models use the ASTNet backbone [42] and follow the training configuration for ShanghaiTech, as described in the original paper. In addition to the configuration-specific models, we include a universal model trained on the combined source data from all configurations. This allows us to evaluate whether a universal model can match or exceed the performance of specialized models under identical resource constraints. For evaluation, all models from the zoo were used off-the-shelf, kept frozen, and uniformly applied to each target dataset without task-specific fine-tuning. Each model encodes the target data into feature representations, which are then used to perform transferability assessments. These assessments rank the models by their adaptability to the target domain. Importantly, in alignment with real-world surveillance constraints, the source data is assumed to be inaccessible during the transferability evaluation process.

The RINN zoo is instantiated with M=20 networks, a value chosen to balance computational cost and ranking stability, as confirmed by the ablation study in Section 4.4. Each RINN adopts the X3D-M backbone [43] from PyTorchVideo (v0.1.5) [44], initialized with randomly sampled weights instead of pretrained checkpoints. For the label-level score $S^{L}$, we reduce X3D’s output layer to 20 classes. Following [20], we train a single fully connected layer to map the pretrained model’s representation $f_{n}\left(X\right)$ to the pseudo labels generated by the RINN, $c^{r_{m}}\left(r_{m}\left(X\right)\right)$. This layer was trained using standard procedures: the learning rate was set using a learning rate range test, and an early stopping criterion on a held-out validation set determined the number of epochs. An exhaustive hyperparameter search was not conducted for this component, as the $S^{L}$ score’s role is to serve as a baseline demonstrating the limitations of classifier-dependent methods. This instability provides the core motivation for our main contribution, the $S^{E}$ score, which is independent of such a classifier. For the embedding-level score $S^{E}$, which directly compares feature embeddings, we remove the final classification layer of X3D. Similarity is computed using minibatch-CKA, adapted from the open-source PyTorch (v2.4.0) implementation by Ristori [45]. The resulting RINN zoo is reused across all target datasets without any fine-tuning. Consistent with real-world surveillance constraints, no target annotations are accessed during the transferability assessment.

### 3.3. Datasets

**Figure 4:**
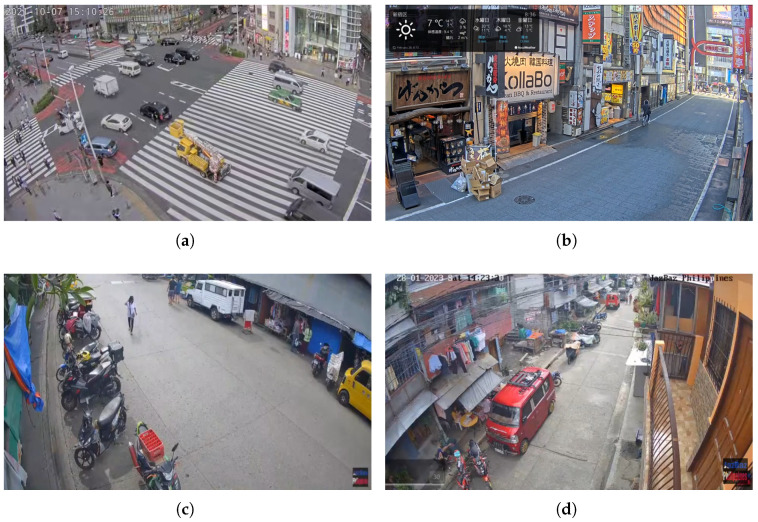
Illustration of four source videos used in training the pretrained model zoo, highlighting the diversity in scene layout and viewing conditions. (**a**) A high-angle camera overlooking a busy intersection with dense traffic and small-scale objects. (**b**) A horizontal view of a pedestrian shopping street with frequent occlusions. (**c**) A roadside market with moderate activity. (**d**) A horizontal view in a domestic neighborhood with low traffic density and residential surroundings. Note that we also include the Mixed model, which is trained on all these four cameras, in the model zoo.

The source data used to train the models in the pretrained model zoo consist of four real-world surveillance videos, captured by cameras positioned at distinct locations with varying viewpoints. These video streams, sourced from live surveillance feeds in Tokyo and Agdao [14,15,16], are illustrated in Figure 4. In Tokyo, one camera captures a large urban intersection (Figure 4a), while the other monitors a narrow alley lined with bars and restaurants (Figure 4b). In Agdao, the Agdao-Market camera provides a close-up view of a bustling roadside market (Figure 4c), whereas Agdao-Street captures a quieter residential street scene (Figure 4d). Temporally, while Tokyo Street [15] covers a short period of night time, the other three datasets are recorded during the daytime. Context-wise, the urban and market scenes (Tokyo Intersection [14], Tokyo Street [15], and Agdao Market [16]) are characterized by high object density and complex layouts, leading to severe and frequent occlusions. Viewpoints range from high-angle, which reduces object scale, to eye-level, where scale can vary dynamically.

For the target data, we use three publicly available datasets: Street Scene [17], NWPU Campus [18], and Urban Tracker [19]. Street Scene and NWPU Campus are designed for anomaly detection. Street Scene contains videos captured from a fixed camera viewpoint, is composed of short, discontinuous clips, and includes 205 annotated anomaly events spanning 17 categories. NWPU Campus includes footage from 43 different cameras, covering 28 distinct anomaly classes. Based on these annotations, both datasets are used for evaluating anomaly detection and event classification tasks. For anomaly detection, we adopt frame-level AUC as the evaluation metric, while event classification is evaluated using instance-level mAP. Since the original annotations only indicate abnormal frames, we manually map each target video into 1 of the 17 or 28 predefined anomaly categories described in the respective papers. Urban Tracker, originally developed for object tracking, comprises five video sequences. We utilize four of them, specifically *Sherbrooke*, *Rouen*, *St-Marc*, and *Atrium*, and exclude *René-Lévesque* due to excessive downscaling that renders objects too small for reliable detection. As the dataset does not provide a predefined train–test split, we follow the protocol in [46], assigning the first 80% of frames for training and the remaining 20% for testing. Since Urban Tracker provides object-level descriptions but not formal semantic classes, we categorize objects based on their free-text descriptions. For object tagging, we evaluate performance using frame-level mean average precision (mAP). Evaluations across these datasets are conducted in accordance with the type of annotation available. All three target datasets were recorded during the daytime and originate from geographic regions (China, North America/Europe) distinct from the source domains. A summary of the datasets used in our experiments is presented in Table 1. Further details can be found in their respective original publications.

### 3.4. Baseline Models and Evaluation Protocols

Very few methods have been developed for transferability assessment under the source-free, label-free setting. Most existing approaches focus on direct domain adaptation rather than explicitly evaluating transferability. In this work, we primarily compare our two proposed transferability metrics, $S^{L}$ and $S^{E}$, against Uncertainty Distance (UD) [26] and Ensemble-based Validation (EnsV) [27], a state-of-the-art method in model selection. Note that EnsV [27] relies on the pretrained classifiers to generate the proxy-groundtruth for model ranking. Since our source models are feature extractors, they cannot be used with EnsV directly. Thus, to adapt EnsV as a baseline, we implement a two-step process. For each source model, we first apply a K-Means clustering algorithm to its output embeddings to generate a set of k distinct pseudo-classes. Subsequently, the label assigned to each embedding is determined by its nearest cluster centroid. To ensure a fair and controlled comparison against our own label-level baseline ($S^{L}$), we set the number of clusters to k = 20, matching the output dimensionality of the $S^{L}$ classifier.

For object tagging and event classification, we attach a fully connected layer on top of each frozen pretrained model, following the protocol in [20]. This layer is fine-tuned on the training set of the target domain, and task accuracy is used as the ground truth to assess the quality of the transferability rankings. To validate whether the estimated model transferability aligns with actual model performance, we report the Kendall’s τ score [47]. Kendall’s τ ranges from −1 (complete disagreement) to 1 (perfect agreement), with 0 indicating no correlation, to quantify alignment between estimated and ground-truth rankings. It is worth noting that, given only five models, Kendall’s τ is highly sensitive: even a single position shift can noticeably affect the score. Similarly, for anomaly detection, we follow the evaluation protocol in [20], using Kendall’s to measure the correlation between estimated rankings and actual performance.

## 4. Results and Discussion

**Figure 5:**
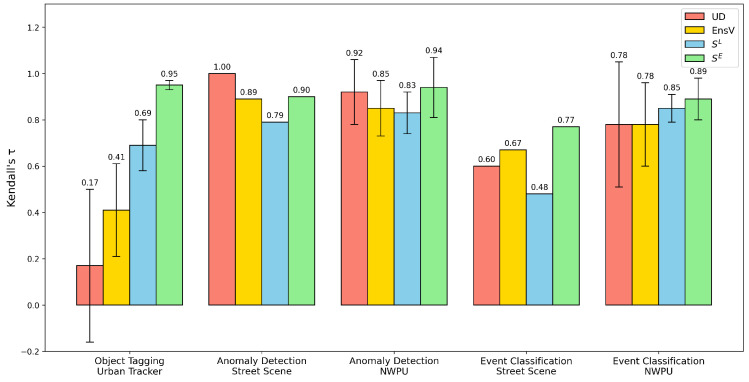
A comparison of transferability assessments. The bar chart shows the mean Kendall’s τ correlation between the rankings produced by each method (UD, EnsV, $S^{L}$, and $S^{E}$) and a proxy ground-truth ranking. The evaluation is performed across five distinct downstream task–dataset combinations. Bars represent the mean performance, and the error bars indicate the standard deviation across multiple camera views where applicable. Higher τ values signify better performance. Our proposed embedding-level score, $S^{E}$, consistently achieves the highest correlation. For the exact numerical values, please refer to Table 2.

In the following sections, we evaluate our transferability estimation approach on the tasks of anomaly detection, object tagging, and event detection. The performance of our method is compared against other baselines (UD, EnsV), with the results visualized in Figure 5 and the precise numerical scores reported in Table 2. We then perform an ablation study to investigate the trade-off between transferability prediction performance and computational cost by varying the number of RINN’s in our approach.

### 4.1. Object Tagging

We evaluate performance on the object tagging task using the Urban Tracker dataset, which includes four diverse camera scenes. As shown in Table 2, $S^{E}$ achieves the highest Kendall’s τ (0.95 ± 0.02), indicating strong and consistent alignment with the ground truth ranking. In contrast, UD yields a much lower correlation (0.17 ± 0.33), suggesting poor reliability in this task. While EnsV provides a more competitive baseline (0.41 ± 0.20), its performance is still notably lower than $S^{E}$, indicating that our method’s direct comparison of embedding structures is more effective than relying on an ensemble’s prediction consensus when a significant task shift occurs. Among the four methods, $S^{E}$ also demonstrates the most stable performance, likely due to its embedding-level comparison, which better preserves structural information from the target data. By contrast, $S^{L}$ relies on a randomly initialized classifier to map RINN features to scalar outputs, making it more susceptible to instability from weight initialization and information compression.

UD’s poor performance merits further examination. While Kendall’s τ is naturally sensitive with only five models, the primary limitation lies in UD’s core assumption: that low model uncertainty on a pretext task (frame reconstruction) correlates with downstream task performance. This assumption fails in object tagging, leading UD to collapse score ranges and obscure meaningful distinctions between models. For instance, in the Rouen scene, UD misidentifies the best-performing model, underscoring how pretext task uncertainty can misrepresent object-level difficulty. Interestingly, UD performs better in tasks where pretext and target objectives are more aligned (see Section 4.2). EnsV is also affected by this fundamental task misalignment, as its clustering is based on the same pretext task embeddings. However, its core mechanism, the reliance on a “joint agreement” from the ensemble, is a double-edged sword. On one hand, it provides a stabilizing effect that filters out noise from poorly performing models, which explains its more competitive ranking compared to UD in scenes like St. Marc. On the other hand, this same property often leads to tied or nearly identical scores among the top-competing models, making the final ranking ambiguous and potentially suboptimal.

**Figure 6:**
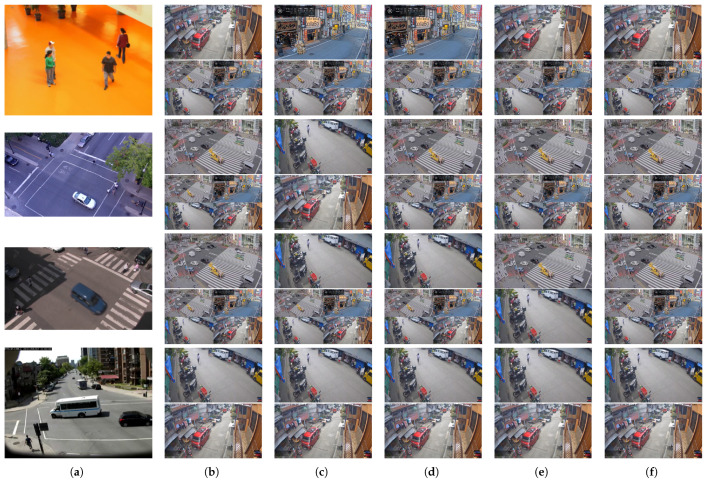
Scene-level qualitative comparison of model selection for object tagging on Urban Tracker. (**a**) shows example frames from the four target scenes: Atrium, St. Marc, Rouen, and Sherbrooke (top to bottom). (**b**–**f**) present the top and second model predictions (top and bottom rows per scene) selected by different methods: (**b**) FCN w/Label (ground truth); (**c**) UD; (**d**) EnsV; (**e**) $S^{L}$; and (**f**) $S^{E}$. Each image shown is an example frame from the original source dataset that the corresponding selected model was trained on. This allows for a visual inspection of the potential domain shift between the selected model’s origin and the target scene. If the *Mixed* model (trained on all four distinct source datasets) is selected, it is represented by a grid of four distinct images, each an exemplar from one of its constituent source datasets. This figure highlights the agreement or divergence in the types of source models selected by the different methods and by the oracle.

To further validate these observations, Figure 6 provides a scene-level comparison of selected models. $S^{E}$ consistently identifies the top-performing model across all scenes. Although UD occasionally agrees with $S^{E}$, even small misorderings, such as those seen in Atrium, St. Marc, and Rouen, can drive Kendall’s τ to zero. These failures typically arise when UD assigns nearly identical scores to its top three models, making its ranking vulnerable to minor fluctuations. Meanwhile, $S^{L}$ and $S^{E}$ show strong agreement, diverging only in the second-best model for Rouen. These results emphasize the value of analyzing the score distribution itself, especially when competing models are closely matched, rather than relying solely on rank-based metrics.

The consistent performance of $S^{E}$ reinforces its independence from output uncertainty and its robustness under task misalignment. Finally, the *Mixed* model (visualized within that cell as a grid of four images), which is trained on footage from all four sources, never secures first place in any scene, highlighting the practical benefit of maintaining a compact model zoo of camera-specific encoders rather than relying on a single “universal” model when computational resources allow.

### 4.2. Anomaly Detection

**Figure 7:**
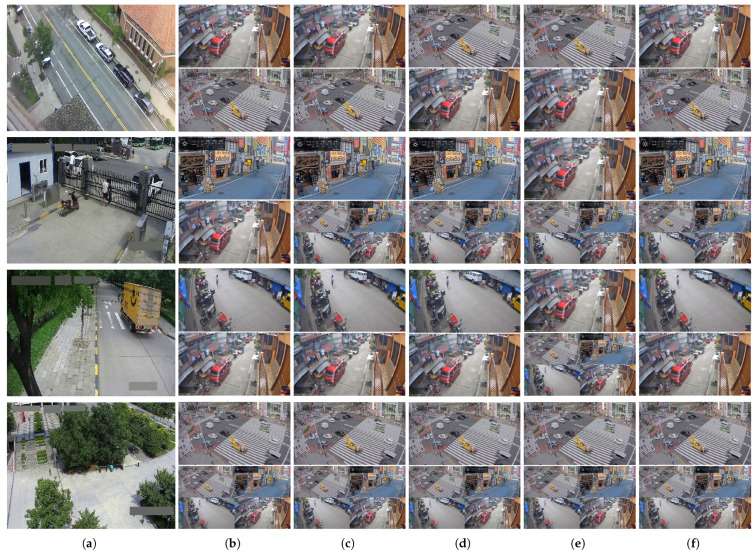
Scene-level qualitative comparison of model selection for anomaly detection on Street Scene and NWPU Campus. The top row shows results from the Street Scene dataset, while the subsequent rows show representative examples from different scenes within the NWPU Campus dataset (D01, D03, D48). (**a**) shows example frames from the four target scenes. (**b**–**f**) present the top and second-best model predictions (top and bottom rows per scene) selected by different methods. (**b**) FCN w/Label (ground truth); (**c**) UD; (**d**) EnsV; (**e**) $S^{L}$; (**f**) $S^{E}$. Each cell shows the prediction of the model ranked best (**top**) and second-best (**bottom**) by the corresponding method, highlighting agreement or divergence with the oracle selection. For detailed quantitative Kendall’s τ scores across all scenes, please refer to Table 2.

The models in the model zoo were originally trained for anomaly detection. We now evaluate how well our transferability assessment methods predict their performance on the same task across new environments. For each pretrained model, we apply a one-class SVM (OCSVM) to its extracted feature representations and compute the AUC score as the performance metric. Since $S^{E}$ is derived from Equation (3), we directly report ${\mathrm{CKA}}^{mb}$ values, which range from 0 to 1. The resulting Kendall’s τ values for Street Scene and NWPU Campus are summarized in Table 2. Qualitative insights into the scene-level model selections are further illustrated in Figure 7. Across most target scenes, all four methods, UD, EnSV, $S^{L}$, and $S^{E}$, achieve τ values above 0.7, indicating strong correlation with the actual model rankings despite the absence of source data or target labels. This confirms the effectiveness of our approach in identifying well-suited models for adaptation. Notably, $S^{E}$ consistently outperforms $S^{L}$ in ranking stability. As also observed in the object tagging task (Section 4.1), this difference stems from the instability of the randomly initialized classifier in $S^{L}$, which often yields imbalanced predictions. In contrast, $S^{E}$ benefits from its embedding-level comparison via ${\mathrm{CKA}}^{mb}$, offering more stable and reliable estimates. This finding is further corroborated by the ablation study in Section 4.4. UD also demonstrates significantly stronger performance on anomaly detection compared to object tagging. In particular, it achieves perfect alignment (τ=1) on Street Scene, outperforming both $S^{L}$ and $S^{E}$. The strong performance of both baselines is expected, as they both leverage the alignment between the models’ original pretext task and the anomaly detection objective. UD’s success is a direct result of this, as it measures uncertainty on the relevant pretext task. EnsV’s competitive ranking performance is also rooted in this alignment between the pretext task and the anomaly detection objective, as the meaningful embeddings produce effective clusters. The final ensemble further provides additional stability by aggregating these predictions. However, Street Scene involves a single fixed viewpoint and may not generalize well to more complex surveillance settings. A more comprehensive evaluation is provided by NWPU Campus, which contains 43 distinct camera perspectives.

On NWPU, both UD and EnsV maintain competitive performance, achieving a higher mean τ and lower variance compared to its object tagging results. It is worth mentioning that EnsV’s performance is slightly more stable compared to UD, due to its characteristic of ensembling model predictions. Nonetheless, $S^{E}$ still secures the highest overall mean τ, with lower sensitivity to scene variability. In contrast, $S^{L}$ remains unstable, exhibiting high variance across scenes, further emphasizing the limitations of classifier-based methods in unsupervised settings and the robustness of embedding-based comparisons.

To further visualize these trends, Figure 7 presents scene-level model selections across NWPU Campus and Street Scene. Figure 7a shows example frames from the target scenes, while Figure 7b–f illustrate the top two models identified by each method. UD and EnsV generally align with the ground truth, deviating in NWPU-D01, where it misorders the second-best model, likely due to minimal differences in the scores. Interestingly, $S^{E}$ also agrees with UD and EnsV in this case, suggesting some ambiguity in the ground-truth ranking. By contrast, $S^{L}$ exhibits greater instability, with inconsistent top-three rankings, underscoring its sensitivity to classifier initialization and output imbalance.

### 4.3. Event Classification

We conclude our per-task evaluation with event classification, where each anomaly instance is assigned a semantic label, as defined in [18]. As shown in Table 2, the performance of both UD and EnsV drop considerably compared to their results on anomaly detection, particularly in the Street Scene dataset. In contrast, both $S^{L}$ and $S^{E}$ maintain stable performance across tasks, with $S^{E}$ consistently achieving the highest Kendall’s τ overall.

This discrepancy is especially notable because all four transferability metrics, namely UD, EnsV, $S^{L}$, and $S^{E}$, are computed without access to target labels and are therefore invariant to task changes. The only component that changes across tasks is the oracle baseline (FCN), which is retrained using event-level annotations instead of anomaly labels. Consequently, the ground-truth ranking of model performance shifts, even though the underlying target data remains the same.

**Figure 8:**
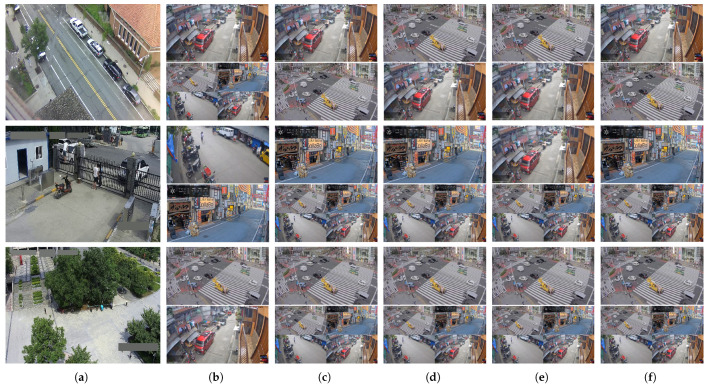
Scene-level qualitative comparison of model selection for event classification on Street Scene and NWPU Campus. The top row shows results from the Street Scene dataset, while the subsequent rows show representative examples from different scenes within the NWPU Campus dataset (D01, D48). (**a**) shows example frames from the four target scenes. (**b**–**f**) present the top and second-best model predictions (top and bottom rows per scene) selected by different methods. (**b**) FCN w/Label (proxy ground truth); (**c**) UD; (**d**) EnsV; (**e**) $S^{L}$; (**f**) $S^{E}$. Each cell shows the prediction of the model ranked best (top) and second-best (bottom) by the corresponding method, highlighting agreement or divergence with the oracle selection. For detailed quantitative Kendall’s τ scores across all scenes, please refer to Table 2.

To explore the implications of this task shift, Figure 8 highlights three representative scenes where the top-ranked model by FCN changes between anomaly detection and event classification. Notably, both $S^{L}$ and $S^{E}$ maintain consistent selections, showing resilience to the shift in downstream objectives. In contrast, both UD and EnsV are more sensitive to these shifts. For example, in the first row (Street), UD ranks the second-best model third, which is similar to $S^{L}$ and $S^{E}$ and and is due to only minor differences in transferability scores. However, in the second (D01) and third (D48) rows, UD misranks the second-best model as fifth, indicating a sharper deviation from the ground truth. EnsV on the other hand, similar to object tagging, generates a tied rank when the task drifts. These cases underscore the vulnerability of UD and EnsV when their pretext assumption, uncertainty as a proxy for downstream performance, breaks under task misalignment, while embedding-based methods like $S^{E}$ remain more robust.

Together, these results illustrate that task changes, even without altering the input data, can lead to significant divergence in model rankings. Methods like $S^{E}$ that rely on structural representation comparison rather than output uncertainty or classifier initialization offer more consistent transferability assessments under such shifts, making them particularly suitable for task-agnostic deployment in smart surveillance systems.

### 4.4. Ablation Study

**Figure 9:**
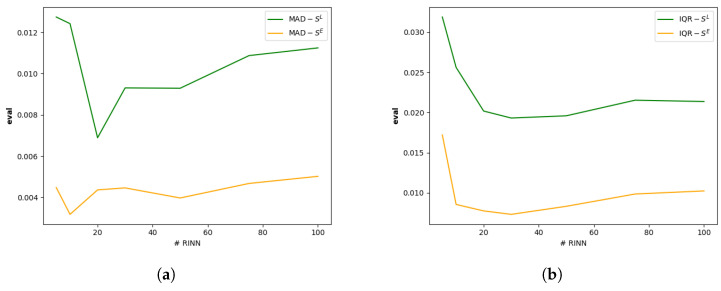
Impact of the number of RINNs used. Shown with (**a**) MAD and (**b**) IQR; the lower the more stable.

While increasing the number of RINNs can improve the reliability of transferability assessments, it also raises computational costs. Therefore, it is essential to strike a balance between assessment stability and computational efficiency. To analyze this trade-off, we randomly initialized 100 RINNs with identical architectures and applied them to the Urban Tracker dataset to compute both $S^{L}$ and $S^{E}$. We then evaluated subsets of the top {5,10,20,30,50,70,100} RINNs and measured the stability of their scores using two statistical metrics: Interquartile Range (IQR) and Median Absolute Deviation (MAD). IQR quantifies the spread between the first and third quartiles, while MAD measures the median of the absolute deviations from the median. Both are less sensitive to outliers than standard deviation, making them suitable for assessing stability in the presence of noisy or highly variable scores. Lower values in either metric indicate greater stability across RINNs. The results, shown in Figure 9, illustrate how stability varies with the number of RINNs, helping to identify an optimal configuration that balances reliability and efficiency.

For both $S^{L}$ and $S^{E}$, we observe that beyond a certain number of RINNs, increasing the ensemble size yields diminishing returns in performance stability. The results indicate that using 20 RINNs provides a good trade-off between reliability and computational efficiency. Additionally, the stability curve for $S^{E}$ demonstrates that the embedding-level assessment is consistently more robust than the label-level counterpart, reinforcing its suitability for resource-constrained deployment.

## 5. Conclusions and Future Work

We address the challenge of identifying the most adaptable pretrained model for source-free, label-free domain adaptation in smart surveillance, where neither the original training data nor target annotations are accessible. Universal models often fall short of the performance achieved by camera-specific models, and only a limited number of transferability assessment methods exist. Among them, approaches like uncertainty distance rely on the questionable assumption that a model’s uncertainty on one task reliably predicts its performance on another. In the absence of labeled target data, effective transferability assessment requires a task-agnostic reference embedding space; this motivates our use of ensembles of randomly initialized neural networks (RINNs) to avoid bias introduced by pretrained representations. We propose a novel and effective framework for transferability estimation in source-free unsupervised settings, specifically tailored to the unique demands of smart surveillance systems, which are characterized by heterogeneous camera configurations and pronounced domain shifts. By leveraging RINNs as unbiased feature extractors, our approach mitigates both structural and task-specific biases inherent in conventional pretrained models. This enables task-agnostic, model-independent transferability assessment. Additionally, our embedding-level metric reduces the computational overhead associated with pseudo label-based approaches, making the method scalable for large surveillance deployments. Empirical evaluations on real-world surveillance datasets demonstrate the practical utility of our method. Specifically, our embedding-level score $S^{E}$ achieved strong Kendall’s τ correlations with ground-truth model rankings across multiple downstream tasks, generally performing comparably to or outperforming other source-free assessment methods. For instance, $S^{E}$ obtained values of 0.95 ± 0.02 for object tagging on Urban Tracker, 0.94 ± 0.13 for anomaly detection on NWPU Campus, and 0.89 ± 0.09 for event classification on Street Scene. This consistent performance indicates that our framework can reliably identify the most adaptable pretrained models, even when the specific downstream task is unknown and both source data and labeled target data are unavailable. These quantitative findings highlight the value of our approach in challenging real-world source-free unsupervised scenarios, particularly in privacy-sensitive and resource-constrained smart city environments.

### 5.1. Limitations

Despite the positive evaluation from the conducted experiments, this work has several limitations that needed to be discussed.

First, a primary limitation arises from the symmetry of minibatch-CKA. We proposed that a large ensemble of RINNs approximates a comprehensive, information-rich embedding space for the target data. The ideal pretrained model, therefore, is one whose representations are a large subset of this embedding space. Our goal is to measure the extent of this informational overlap. However, minibatch-CKA is symmetric which indicates the degree of overall alignment between the two embedding spaces rather than measuring this one-way, subset relationship. This is compounded by potential redundancy within the RINN ensemble. Since our method does not explicitly decorrelate the features from different RINNs, it risks overestimating certain information and impairing the model ranking.

Second, our experimental validation is limited in scope. The evaluated tasks are classification-related tasks and its efficacy for other surveillance applications, such as anomaly event localization, semantic segmentation, and crowd counting, remains unverified. Furthermore, the experiments were conducted on static datasets, and the analysis does not account for temporal dynamics or concept drift, where data distributions evolve over time.

Third, the study is bounded by the limitations and biases of the datasets used. The source datasets are mostly recorded during the daytime. Geographically, these datasets are biased to specific urban and residential environments, which presents a realistic and challenging transferability scenario when assessing models on target data from different global contexts. The target datasets also possess unique constraints: Street Scene [17] is limited by a single, fixed viewpoint and short, discontinuous clips; NWPU Campus [18] has a known data imbalance and limited object interaction; and Urban Tracker [19] contains non-standard annotations and variable technical quality. Despite these limitations, by intentionally evaluating our method across this diverse and complementary set of target domains, we have subjected our approach to a more holistic and stringent test of its robustness. While each individual experiment is bounded by the nature of its data, the overall experiments provide broader evidence of our method’s applicability across varied conditions.

Finally, practical and computational factors present further considerations. While the method avoids the cost of full fine-tuning, it incurs its own practical costs. The need to generate and store RINN weights creates a non-trivial memory footprint and computational overhead. This paper does not include a formal cost–benefit analysis comparing this assessment overhead to other lightweight baseline strategies.

Acknowledging these boundaries is crucial for contextualizing our findings and provides a clear roadmap for future research.

### 5.2. Future Work

In future work, our research direction will focus on several key aspects, focusing on methodological refinement, expanded empirical validation, and deeper theoretical analysis.

First, we will refine our core assessment method. This involves exploring asymmetric embedding similarity metrics to better estimate directional information transfer between representations. We will also investigate the impact of different RINN architectures and initialization strategies to more effectively construct the ensemble. Although RINNs require no training, they possess a non-trivial memory footprint. While the current assessment was conducted on resource-rich cloud infrastructure, developing a more memory-efficient implementation is crucial for large-scale deployment. Furthermore, our current approach is a one-step process in assessing the model transferability. To further adapt the selected model to target data, a more efficient model adaptation strategy must be investigated.

Second, we will expand the scope of our experimental validation. This includes applying the method to non-classification tasks (e.g., semantic segmentation), extending the analysis to streaming data to assess performance against temporal drift (e.g., changes in lighting and weather), and evaluating against target datasets from more diverse geographical and temporal contexts to ensure robustness. This expansion also includes a plan to expand the diversity of the model zoo. The current study utilized source models from the video surveillance domain; a comprehensive investigation using models from disparate domains (e.g., general image or video datasets such as ImageNet or Kinetics) will be necessary to rigorously test the framework’s generalizability.

Third, we will conduct a comparative analysis of the use of pretrained embeddings and RINN embedding. Our method avoids reliance on pretrained embeddings; this design is motivated by observations in prior work and our own empirical findings suggesting that such embeddings do not generalize reliably when the downstream task differs from the pretext task. This issue is particularly relevant in smart surveillance scenarios, where source models are often trained for general purposes, while the downstream task (e.g., anomaly detection, event classification, or object tagging) is not fixed at deployment. While our approach demonstrates greater consistency, it should be understood as a robust alternative to, rather than a direct solution for, the poor generalization of pretrained embedding-based methods under task drift. Future work could conduct a theoretical analysis of how pretrained embedding-based methods behave under task uncertainty, and whether RINN embedding-based assessment can adapt such reliance when the task alignment is known and further provides more stable guidance.

## Figures and Tables

**Table 1:** A summary of the source and target datasets used in our experiments. This table details the role of each dataset, its core scene characteristics, and its technical specifications.

Dataset	Scene Characteristics				Specifications		
	Environment	Density	View	# Cams	Frames	Resolution	fps
Tokyo Intersection [14]	Urban Intersection	Dense	High-level	1	432,000	1920 × 1080	30
Tokyo Street [15]	Shopping Street	Moderate	Eye-level	1	324,000	1920 × 1080	30
Agdao Market [16]	Roadside Market	Dense	Eye-level	1	432,000	1920 × 1080	30
Agdao Residential [16]	Residential Street	Sparse	Mid-level	1	432,000	1920 × 1080	30
Street Scene [17]	Two-lane Street	Sparse	High-level	1	202,545	1280 × 720	15
NWPU Campus [18]	University Campus	Varies	Mixed	43	1,466,073	Various	25
Urban Tracker [19]	Mixed	Dense	Mixed	4	600–4540	Various	25–30

**Table 2:** Detailed Kendall’s τ scores for each transferability assessment. Results are reported as mean (±) standard deviation. No deviation is reported for Street Scene as it uses a single camera setup.

Task	Object Tagging	Anomaly Detection		Event Classification	
Dataset	Urban Tracker	Street Scene	NWPU	Street Scene	NWPU
**UD**	0.17 ± 0.33	1.00	0.92 ± 0.14	0.60	0.78 ± 0.27
**EnsV**	0.41 ± 0.20	0.89	0.85 ± 0.12	0.67	0.78 ± 0.18
$S^{L}$	0.69 ± 0.11	0.79	0.83 ± 0.09	0.48	0.85 ± 0.06
$S^{E}$	0.95 ± 0.02	0.90	0.94 ± 0.13	0.77	0.89 ± 0.09

## Data Availability

The Street Scene dataset can be downloaded from https://zenodo.org/records/10870472, Zenodo (accessed on 17 June 2025). The NWPU Campus dataset can be downloaded from https://campusvad.github.io/, NWPU (accessed on 17 June 2025). The Urban Tracker dataset can be downloaded from https://www.jpjodoin.com/urbantracker/dataset.html, Urban (accessed on 17 June 2025). The Tokyo and Agdao datasets are publicly available on Youtube, with the following link: https://www.youtube.com/watch?v=xiLF6PmFZP4, Tokyo Shinjuku Live (accessed on 17 June 2025), https://www.youtube.com/watch?v=bq7jWW7dfws, Shinjuku Kabukicho (accessed on 17 June 2025), and https://www.youtube.com/watch?v=NxG2Hor92DE, PHILIPPINES Street View quad Camera, Agdao, Davao City (accessed on 17 June 2025).

## Abbreviations

    The following abbreviations are used in this manuscript:

RINN	Randomly Initialized Neural Network
SFUDA	Source-free Unsupervised Domain Adaptation
GDPR	General Data Protection Regulation
